# Frequency-diverse multimode millimetre-wave constant-*ϵ*_*r*_ lens-loaded cavity

**DOI:** 10.1038/s41598-020-78964-1

**Published:** 2020-12-17

**Authors:** M. A. B. Abbasi, V. F. Fusco, O. Yurduseven, T. Fromenteze

**Affiliations:** 1 Centre for Wireless Innovation (CWI), Institute of Electronics, Communications and Information Technology (ECIT), School of Electronics, Electrical Engineering and Computer Science (EEECS), Queen’s University Belfast, Belfast, BT3 9DT UK; 2 XLIM Research Institute, University of Limoges, 87060 Limoges, France

**Keywords:** Energy science and technology, Engineering, Mathematics and computing, Physics

## Abstract

This paper presents a physical frequency-diverse multimode lens-loaded cavity, designed and used for the purpose of the direction of arrival (DoA) estimation in millimetre-wave frequency bands for 5G and beyond. The multi-mode mechanism is realized using an electrically-large cavity, generating spatio-temporally incoherent radiation masks leveraging the frequency-diversity principle. It has been shown for the first time that by placing a spherical constant dielectric lens (constant-*ϵ*_*r*_) in front of the radiating aperture of the cavity, the spatial incoherence of the radiation modes can be enhanced. The lens-loaded cavity requires only a single lens and output port, making the hardware development much simpler and cost-effective compared to conventional DoA estimators where multiple antennas and receivers are classically required. Using the lens-loaded architecture, an increase of up to 6 dB is achieved in the peak gain of the synthesized quasi-random sampling bases from the frequency-diverse cavity. Despite the fact that the practical frequency-diverse cavity uses a limited subset of quasi-orthogonal modes below the upper bound limit of the number of theoretical modes, it is shown that the proposed lens-loaded cavity is capable of accurate DoA estimation. This is achieved thanks to the sufficient orthogonality of the leveraged modes and to the presence of the spherical constant-*ϵ*_*r*_ lens which increases the signal-to-noise ratio (SNR) of the received signal. Experimental results are shown to verify the proposed approach.

## Introduction

Fast and accurate update of the channel state information is critical for a millimetre-wave (mmWave) communication system to work. Towards this end, accurate direction-of-arrival (DoA) estimation is required at the base station (BS). Some of the most widely used DoA estimation techniques include MUSIC algorithm^1–3^, ESPRIT algorithm^4–6^, Capon algorithm^7–9^, and Bartlett algorithm^10,11^. These techniques generally require an array of antennas at the BS along with the multiple radio frequency (RF) electronic systems. These techniques are widely used at low frequencies (covering the sub-6 GHz bands of the 5G^12^ and preceding communication generations). However, at mmWaves, the RF electronics hardware is expensive and a multiplicity of independent channels required to connect each antenna element to the base-band processing units for the DoA estimation algorithms to work, necessitates considerable channel calibration^13^. In addition to this, mmWave antenna arrays have generally a higher number of antennas compared to lower frequency (sub-6 GHz) arrays, and that is because the mmWave radios require an additional array gain to compensate for higher path loss compared to sub-6 GHz wireless systems and additional antennas are required for null filling due to the high directivity of the array antennas^14,15^. Because of these challenges, new techniques and methods are required in the mmWave spectrum which allow fewer antennas and a lower number of RF-chains to support the mmWave DoA estimation. A number of new mmWave antenna hardware design approaches has been investigated recently, e.g.^16–20^, that tried to reduce the cost and complexity of the radio hardware, which is a significant goal of the telecom industry, towards successful mmWave 5G deployment^16^.

Classical DoA techniques require a beam synthesis process at the receiver end by which channel state information is collected by mechanical or electrical beam steering. Mechanical beam scanning is not fast enough to cope with the channel mobility expected in mmWave channels^16^. Electronic beam scanning like the one using phased arrays is applicable^21–23^ but is expensive to implement. The concept of highly directive frequency-diverse antenna apertures have recently received attention as an alternative solution in which the overall hardware cost and complexity is very low compared to fully equipped and fully connected antenna array^24^. This concept is derived from significant research efforts in the computational imaging domain^25–29^ in which 3-dimensional (3D) scene information is encoded in terms of quasi-randomness of the measurement modes (*N*) along a frequency bandwidth. The computation imaging application work in^25–29^ is limited to near-field where the frequency-diverse aperture works as a transmitter and a receiver, however, the DoA estimation problem using highly directive frequency-diverse antenna apertures works purely as a receiver, and the technique is required to work into the far-field. For this application, channel information within a field of view (FoV) in terms of far-field radiation source should be able to be re-constructed from quasi-random measurement modes, in conjunction with the mode-mixing cavity transfer functions and computational techniques, such as the least-square algorithm and matched-filtering in a given bandwidth^30^. A multiplexing technique in the physical layer for microwave imaging for far-field using ultra-wide band (UWB) antenna array is shown in^31^, while the concept of passive multiplexing for imaging was introduced in^32^. It is important to stress that the compressed imaging for channel sounding application has not been demonstrated before. Whereas a preliminary theoretical investigation in this domain was carried out in^24^ with a hypothetical high-Q factor frequency-diverse antenna, in this paper, we demonstrate the first numerical and experimental validation of a computational frequency-diverse cavity-backed metasurface antenna loaded with a lens for channel characterization in the form of a DoA estimation problem.

In the work we now present, we show for the first time that usable results can be achieved even after these theoretical requirements for frequency-diverse antenna are significantly relaxed. A radiating aperture as a transmitter with Luneburg lens is shown in^33^. In the approach we describe in this paper, the radiating aperture of a relatively low Q-factor mode-mixing cavity covered with a high gain constant-*ϵ*_*r*_ lens^34,35^ and is coupled through a curved surface with sub-wavelength holes. This geometric configuration concentrates the radiation intensity in an angular sector in front of the lens-loaded cavity, helping to overcome propagation losses, and additionally requires only a single radio frequency (RF) channel. The compressed signal received at the RF output of the lens-loaded cavity is computationally processed to give DoA estimates of incoming mmWave signal angle(s) of arrival.

The motivation behind our approach can be summarized as follows. Firstly, the known methods of DoA estimation requires an array of antennas with each antenna requiring a separate connection to the base-band processing unit via a dedicated RF chain, resulting in an increased hardware cost especially at the mmWave spectrum. Secondly, the known methods of using mode-mixing cavity (like in^25,26,28,29^) have their core capability limited to near-field microwave imaging. The size of a mode mixing cavity is large in sub-6 GHz 5G frequencies, making it difficult to mount to radio sub-systems and/or base-stations antennas, while the mode-mixing cavity size is practical at mmWave frequencies.

### Contributions

Our approach poses following advantages over the current state of the art. Firstly, the lens-loaded cavity not only generates frequency-diverse modes, but due to the spherical constant-*ϵ*_*r*_ lens operation, enhances the gain of the radiation mask. Secondly, the estimation scheme in our proposed architecture uses spatio-temporal orthogonal modes, which needs only a relatively modest frequency-sweep and a single RF-chain to estimate DoA. For the first time, it is shown that by leveraging the concept of frequency-diversity together with the focusing capability of a spherical constant-*ϵ*_*r*_ lens, the DoA of a plane-wave striking the lens-loaded cavity can be accurately retrieved. Thirdly, with the help of antenna characterization, it is shown that the size of the lens-loaded cavity makes the proposed solution viable for mmWave operation. Fourth, it is shown that the sub-wavelength coupling arrangement used equalizes the radiating energy from the lens-loaded cavity across a wide FoV coverage sector.

## Methods

### System architecture

**Figure 1:**
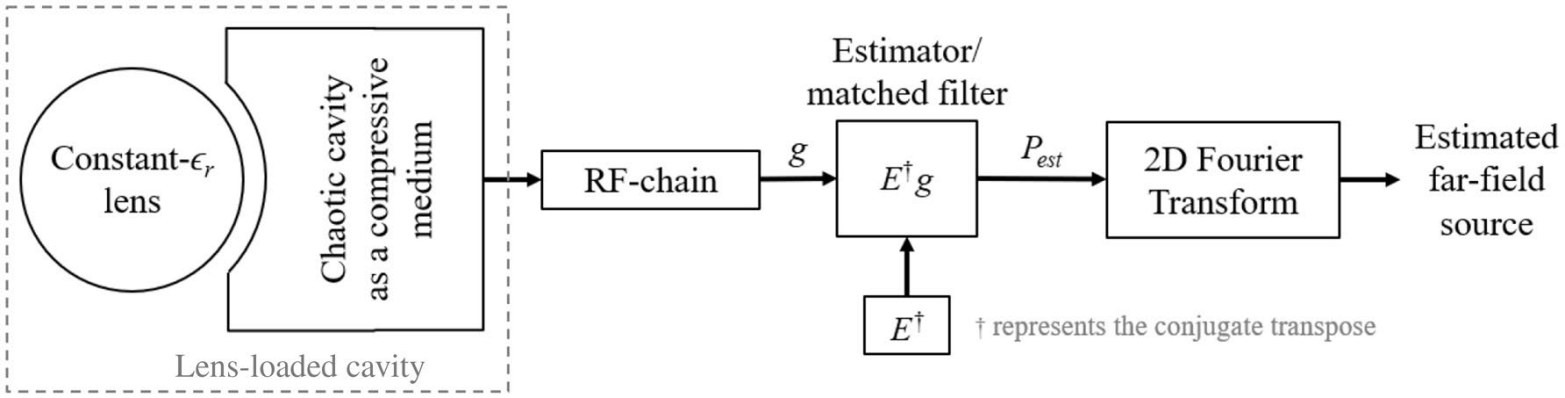
System block diagram with the lens-loaded cavity, RF and signal processing blocks.

**Figure 2:**
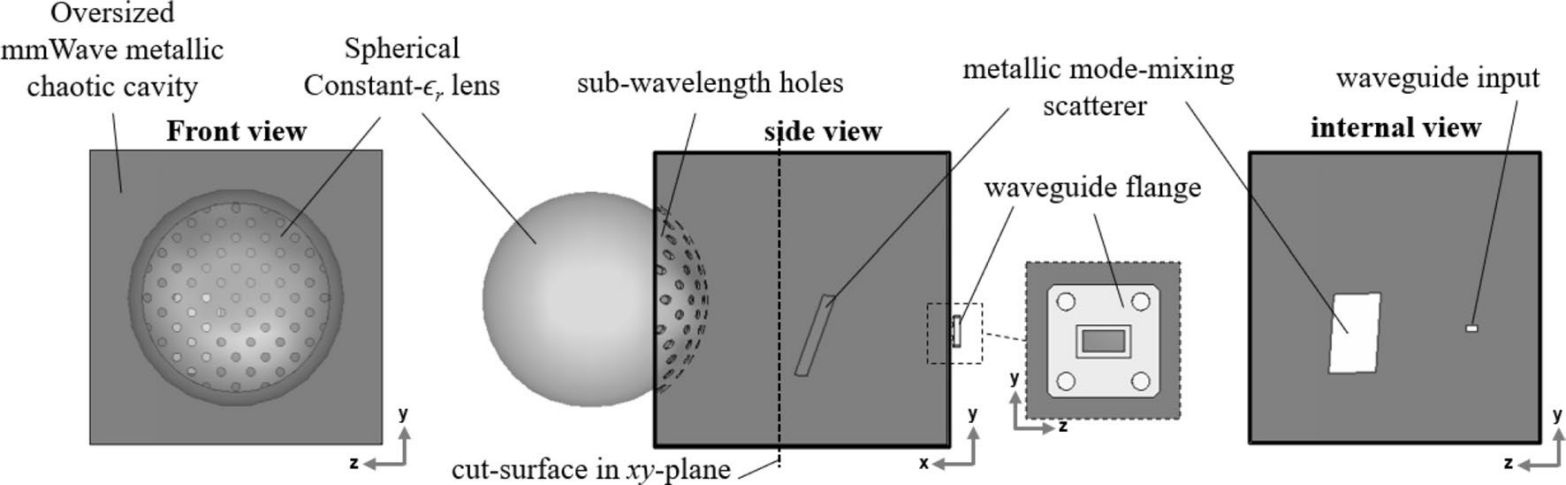
Frequency-diverse multimode millimetre-wave lens-loaded cavity with a front, internal side and internal front elevations.

The presented system architecture requires only a single lens-loaded cavity radiator with a single input/output, and the system block diagram is shown in Fig. 1. The system architecture consists of a lens-loaded cavity connected to an RF chain, which is subsequently connected to a computational unit. The lens-loaded cavity in Fig. 1 comprises of a multi-mode cavity having volume from 15λ × 15λ × 15λ to 18λ × 18λ × 18λ over the frequency range 27–29 GHz air-spaced coupled through a sub-wavelength hole array and a spherical constant-*ϵ*_*r*_ lens having diameter of 12.4 λ at the central frequency of operation, 28 GHz. The structural configuration of the lens-loaded cavity is presented in Fig. 2, which shows the mmWave metallic cavity and the curved radiating surface with sub-wavelength holes having diameter of 0.8 λ at 28 GHz. The centre to centre distance between the consecutive holes on the curved surface is 1.2 λ at 28 GHz and coupling through it follows the principle of extraordinary microwave energy transmission through an array of subwavelength holes^36^. The curved surface follows the circumference of a full spherical constant-*ϵ*_*r*_ lens, while the rear hemispherical surface of the lens is placed 3.5 mm from the hole array. A metallic sheet of size 30 × 52 mm^2^ is placed inside the cavity at an arbitrary location and orientation in order to enhance the mode-mixing capability of the cavity by introducing quasi-random disorderly reflections without the requirement of any external mode-mixing mechanism. This way we can avoid the superposition of wave vectors in the reciprocal space, which will lead to symmetry breaking in the measurement modes. The presence of the mode mixing scatterer between the sub-wavelength hole opening and the input/output channel will result in attenuation of the direct paths between the feed and the lens, which helps in limiting the level of spatial correlation within the cavity. The mmWave chaotic cavity constructed from copper is terminated into a standard WR28 waveguide.

### Lens-loaded cavity

**Figure 3:**
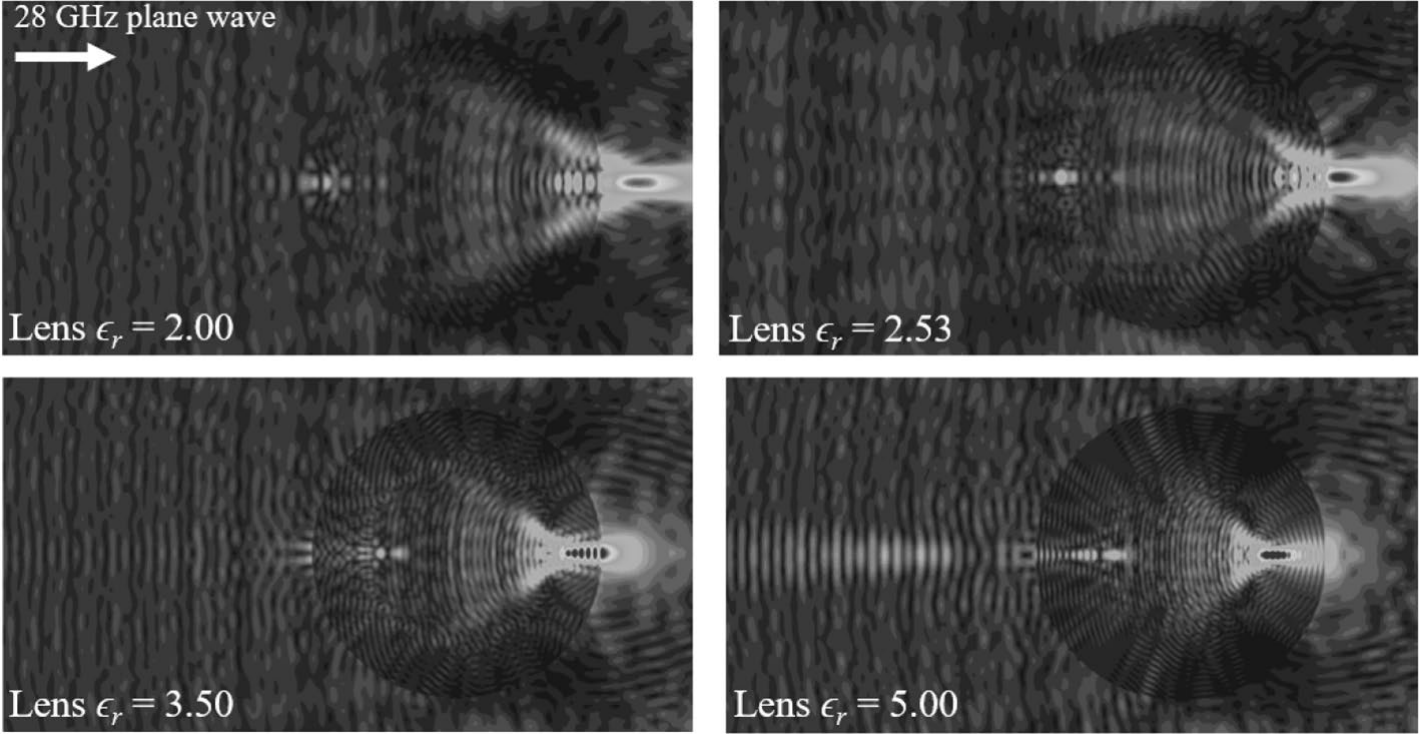
Energy focusing capacity and focal area for the spherical constant-*ϵ*_*r*_ lenses with multiple *ϵ*_*r*_ values.

A spherical constant-*ϵ*_*r*_ lens has a unique property, for a specific range of *ϵ*_*r*_ values, of being able to focus incoming microwave energy to locations outside the lens surface defined by its Petzval curvature^37^. Typically, suitable lens operation is possible for *ϵ*_*r*_ values greater than 2.0 and less than 3.5^34^. The constant-*ϵ*_*r*_ lens's material selection is generally governed by two choices, one is the maximum utilizable lens spherical area and other is the position off the lens where the energy is to be focused to, Fig. 3. In our case, this choice yields *ϵ*_*r*_ = 2.5, with the resulting focal surface 3.5 mm from the spherical constant-*ϵ*_*r*_ lens surface chosen so as to prevent the generation of unwanted reflections between the cavity and the constant-*ϵ*_*r*_ lens as well as minimising energy leakage into the external environment around the lens when a 28 GHz plane wave strikes the lens surface. The material chosen for lens realisation is Rexolite^38^ with *ϵ*_*r*_ = 2.53. This material has low loss tan*δ* ≈ 0.00066, which means that the signal passing through it will suffer significantly low attenuation compared to the gain achieved due to energy focusing capacity. The benefit of using a lens for antenna gain enhancement is reinforced in^34^, while details on the effect of lens material on losses can be found in^39^. Rexolite material is readily machined and highly resistant to moisture absorption and exhibits low dimensional variation due to external environmental influences such as temperature and humidity, thus helping to mitigate long term calibration issues.

Consider now the lens-loaded cavity in Fig. 2 facing towards the + *x*-direction in a Cartesian coordinate system. A plane wave is incident upon the lens-loaded cavity from the FoV along − *x*-direction, *θ* and *φ* represents azimuthal and elevation angles in the incident wave direction. The lens-loaded cavity operates from 27 to 29 GHz and has a single WR28 channel output connected to the RF-chain. The exponential decay associated with the cavity dictates the time domain impulse response $h(t)$ of the lens-loaded cavity. This is proportional to the loaded Q-factor of the structure in Fig. 2 and is given by $Q = \pi f_{0} \tau$^40^, where *f*_0_ is the central frequency, here 28 GHz. The channel impulse response is given by

1 $$h_{m} \left( t \right) = n_{m} \left( t \right)e^{{ - \frac{2t}{\tau }}}$$

where $n_{m} \sim \,N\left( {0,\sigma^{2} } \right)$ when *N* is the normal distribution with 0 mean and $\sigma^{2}$ variance. This suggests that a high Q-factor is desirable since it will enhance a frequency-diverse antenna aperture impulse response $h(t)$, i.e. reduce the correlation between multiple modes^19,24^. The number of useful modes in frequency-diverse antenna apertures is given in^40^ as $N_{\max } = {{QB} \mathord{\left/ {\vphantom {{QB} {f_{0} }}} \right. \kern-\nulldelimiterspace} {f_{0} }}$ where *B* is the bandwidth of operation and *f*_0_ is the centre frequency. The Q-factor of the lens-loaded cavity is calculated to be *Q* = 4636 at 28 GHz using the energy decay profile, simulated in the CST Microwave Studio^41^, using method given in^42^. Hence, the upper bound limit on the number of modes is *N*_max_ is 331 modes. Later it will be shown that we can still characterize the channel within the FoV even when the practical *N* is significantly less than 331 i.e. 41.

It should be noted that, in this work, we use the 27–29 GHz band to demonstrate the application of the lens-loaded frequency-diverse cavity for the entire 28 GHz 5G spectrum. Whereas it is possible that a 5G channel can occupy a smaller bandwidth, the developed lens-loaded cavity can readily generate *N*_max_ = 331 modes. Therefore, for a 5G channel with a smaller bandwidth, the presented technique can readily produce a sufficient number of modes to achieve DoA estimation. For example, considering 5G channel with 500 MHz bandwidth, the developed cavity can produce 66 orthogonal modes, which is above the number of modes that we show is sufficient to achieve high-fidelity DoA retrieval in this work, i.e. 41. Moreover, by further increasing the Q-factor of the antenna, we can further grow the number of frequency-diverse modes sampling the incident plane-wave.

Following the same definition for the characterization plane of a frequency-diverse aperture as given in^24^, we evaluated the mode generation capacity of the lens-loaded cavity first through simulation and then by measurement. The transfer function of a frequency-diverse aperture can be obtained experimentally^28^ or analytically^24^. The radiated field from the lens-loaded cavity can be approximated by creating a metasurface loaded with a number of meta-elements generating repeatable field maps. This is analogous Huygens' metamaterial surfaces^43^ in which electric and magnetic sheet impedances provide the required currents to generate a prescribed radiating wave. The projection of a radiating aperture on a characterization plane can be written as:

2 $$E_{\omega } (\overline{r}) = \int\limits_{r^{\prime}} {m_{\omega } (\overline{r}^{\prime})G_{\omega } (\overline{r},\overline{r}^{\prime})d\overline{r}^{\prime}}$$

where $\overline{r}^{\prime}$ is the coordinates of the characterization plane, $\overline{r}$ on the equivalent aperture plane $m_{\omega } (\overline{r}^{\prime})$ is the radiation of a single meta-element on the antenna aperture, while $G_{\omega } (\overline{r}_{1} ,\overline{r}_{2} )$ is the Green’s function defined as

3 $$G_{\omega } (\overline{r}_{1} ,\overline{r}_{2} ) = \frac{{e^{{ - jk_{0} \left| {\overline{r}_{2} - \overline{r}_{1} } \right|}} }}{{\left| {\overline{r}_{2} - \overline{r}_{1} } \right|}}$$

in which *k*_0_ is the wavenumber. Due to far-field approximation, the magnitude term in Eq. (3) is dropped. The DoA estimation problem is then calculated using far-field projection in the form of:

4 $$p(\theta ,\varphi ) = e^{{ - jk_{0} \left( {y\sin \theta \cos \varphi + z\sin \theta \sin \varphi } \right)}}$$

Note that the calculations in Eq. (4) assume that the far-field source remains constant across the frequency band of operation and we are not correcting any time domain dispersion since the bandwidth is rather narrow. Therefore, the wavenumber *k*_0_ in (4) is fixed at 28 GHz. It should be noted that this is not a fundamental limitation of the presented technique, but rather is an assumption to simplify the mathematical model of the far-field source. This assumption is valid for the scenarios in which coherence intervals > 0.5 ns, which is reasonable for mmWave 5G channels with low terminal mobility^44^. Moreover, given the central frequency, 28 GHz, the frequency variation is limited to ± 1 GHz around 28 GHz, suggesting a maximum of only ± 3.5% fractional bandwidth window centred around 28 GHz. So, for a consistent and normalized plane wave incident on the lens-loaded cavity, the compressed signal at the output port of the lens-loaded cavity after the RF chain (see Fig. 1) can be written in the form,

5 $$g(\omega ) = \int\limits_{{\overline{r}}} {E(\overline{r},\omega )p(\overline{r})d\overline{r}}$$

this is then followed by the DoA estimator using a matched filter reconstruction algorithm.

**Figure 4:**
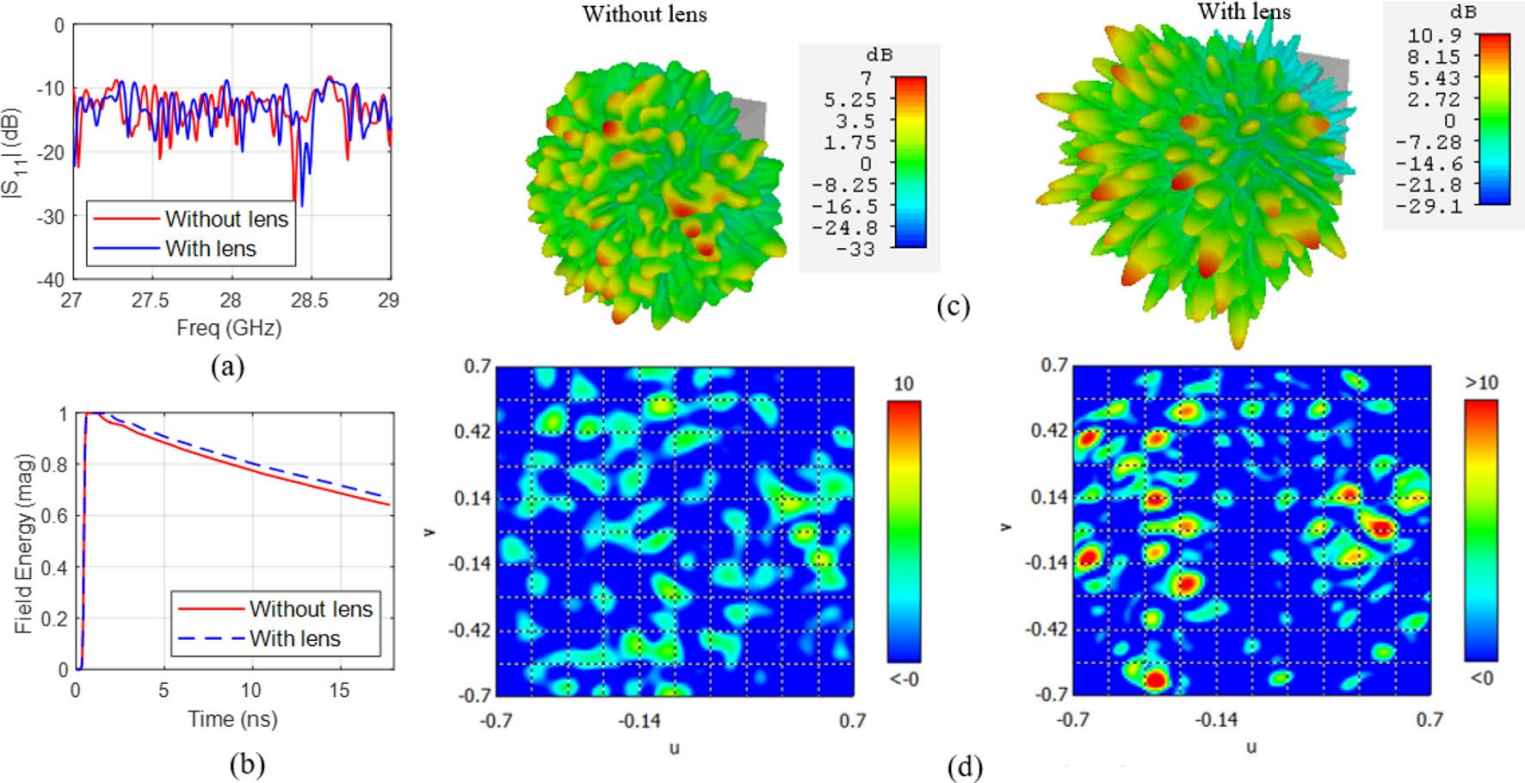
Simulation results of the lens-loaded cavity (**a**) matching, (**b**) energy decay profile and gain with and without the spherical constant-*ϵ*_*r*_ lens (**c**) in the form of 3D radiation pattern, and (**d**) in *uv*-plane at 28 GHz.

**Figure 5:**
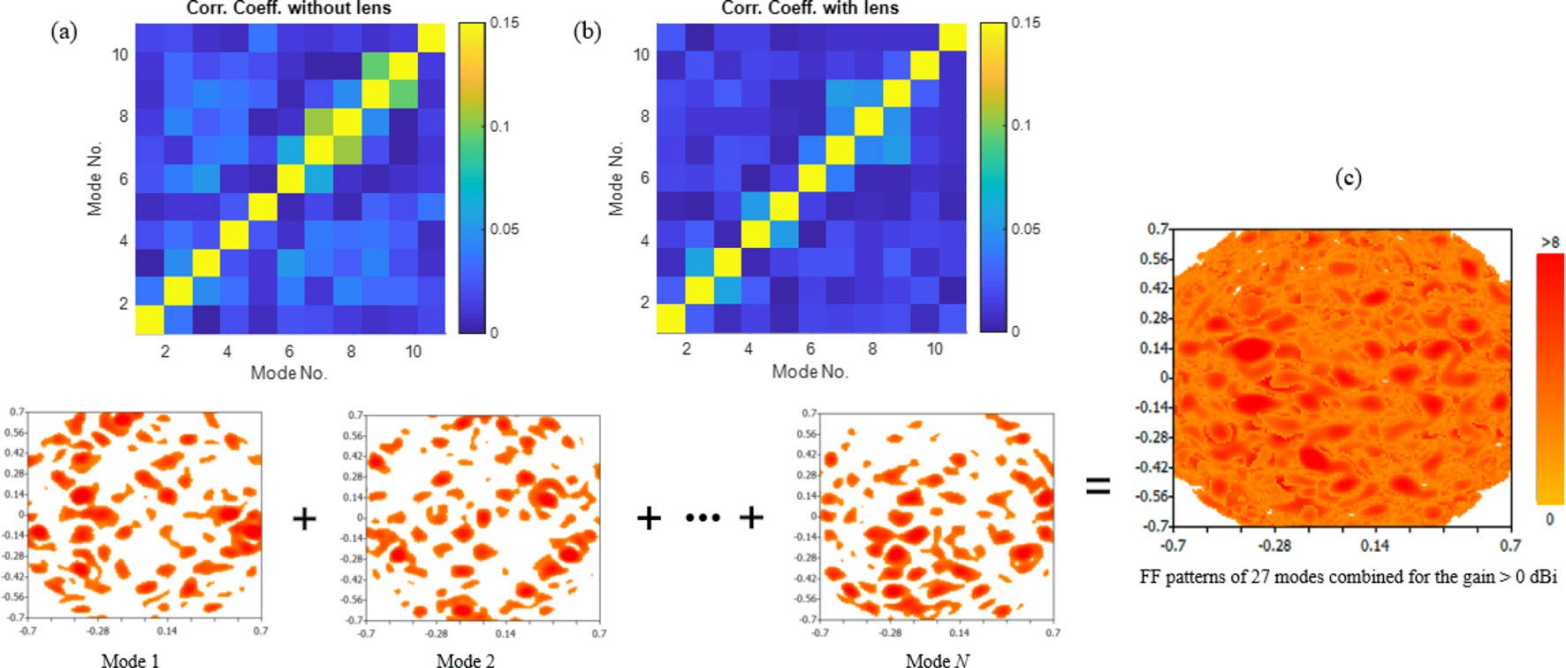
Correlation coefficient of 11 mode patterns (**a**) without lens and (**b**) with lens from 27.5 to 28.5 GHz. (**c**) Superposition of *N* individual mode patterns when the 2D contour plots are clamped for any value of the gain below 0 dB. Superposition of *N* = 21 modes show that the entire coverage of the forward half space FoV is possible using the lens-loaded cavity.

In order to elaborate the contribution of the lens-loaded cavity in the system architecture of Fig. 1, let us consider the effect of input signal excitation at the waveguide port in Fig. 2. The simulation results for this setup at the centre frequency, i.e. 28 GHz, are given in Fig. 4. Resonance modes and the slope of energy decay shown in Fig. 4a,b shows that the design of the lens and coupling scheme used results in high quality antenna matching and resulting modal Q-factor. The benefit of the spherical lens placed in front of the frequency-diverse cavity can be seen Fig. 4c with the lens significantly helping to confine the radiating energy into the FoV along the + *x*-direction. Also, from Fig. 4d, when the far-field is represented in *u*, *v* plane defined as,

6a $$u = \sin \varphi \cos \theta$$

6b $$v = \sin \varphi \sin \theta$$

the sectoral coverage for the cavity with the spherical constant-*ϵ*_*r*_ lens is found to be lower compared to the same cavity without lens. This is as a result of the peak gain value at 28 GHz with lens case being increased to 10.9 dBi from 6.8 dBi without the lens. Over the frequency range of 27 to 29 GHz by using the spherical constant-*ϵ*_*r*_ lens, we can enhance system gain by as much as 6 dB. This enhancement in the gain increases the spatial incoherence between the orthogonal modes, improving the mathematical conditioning of the inverse problem which will be further explained from the measurement results and singular value decomposition (SVD) analysis in the next section. Note that the measurement modes in DoA estimation context means field patterns on the characteristic plan for discrete frequencies within the bandwidth of interest. This is analogous to the measurement modes in microwave imaging^28,30^ in which when the driving frequency of frequency-diverse antenna aperture is varied, the field pattern changes. This leads to a diverse set of measurement modes just by sweeping frequency. From the perspective of a coded aperture^29^, the radiation modes are very different from a regular antenna, in that we essentially use “a multiplicity of sidelobes” to illuminate multiple pixels in the FoV. This is evident in the field plots of Fig. 4c that the “sharper” the beams become, the less likely that they will overlap with the sidelobes of the next mask. By increasing the gain of the sidelobes probing the FoV information, we are sharpening the sidelobes and reducing the overlap between masks. To further elaborate this point, correlation coefficient contours for 11 radiation masks with and without the presence of spherical lens is given in Fig. 5a,b, from where the positive impact of the presence of lens can be observed. As discussed before, for the simplicity of the model we are considering modes on discrete frequencies that are equidistance on the frequency spectrum. Figure 5b also confirms that our approximation is valid since the recorded fields on these discrete frequencies have low correlation. The superposition of the radiation masks for the far-field of first 21 modes that correspond to the frequencies at which the lens-loaded cavity radiates most efficiently are shown in Fig. 5c. The result shows that the entire forward sector within − 60° < *θ* < 60° and − 60° < *φ* < 60° FoV can be sampled with a multiplicity of spatial modal diversity with no shadow zones and with approximately equal power per spatial mode.

## Measurements and results

A proof-of-concept hardware is constructed in which the oversized mmWave chaotic cavity is created using 180 × 180 mm^2^ single sided copper coated substrate sheets. The curved surface with sub-wavelength holes is created using carefully bent copper strips, while sub-wavelength holes of diameter = 5.34 mm are machined using an LPKF Protomat H100 milling machine. A plane copper sheet placed within the cavity is used as a randomly oriented scatterer and metal adhesive tape is used to seal the cavity edges. A spherical constant-*ϵ*_*r*_ lens is created by machining out a solid spherical lens from Rexolite plastic material rod. In addition to the electrical properties mentioned before, other properties of Rexolite material like density = 1.05 g/cm^3^, coefficient of linear thermal expansion 70 × 10^–6^ °C^−1^ and thermal conductivity 0.146 W m^−1^ °C^−1^ makes it an excellent choice for mmWave lens development. The synthesis approach given in^34^ resulted in the Rexolite lens focal point to be at 77.5 mm from the lens spherical origin while the lens diameter is 133 mm (radius 66.5). A Styrofoam holder is machined to offset the lens by 3.5 mm in front of the cavity. A K-type to WR28 converter is connects the lens-loaded cavity at 38 mm and 68 mm from the left bottom corner to a wideband coax input/output.

**Figure 6:**
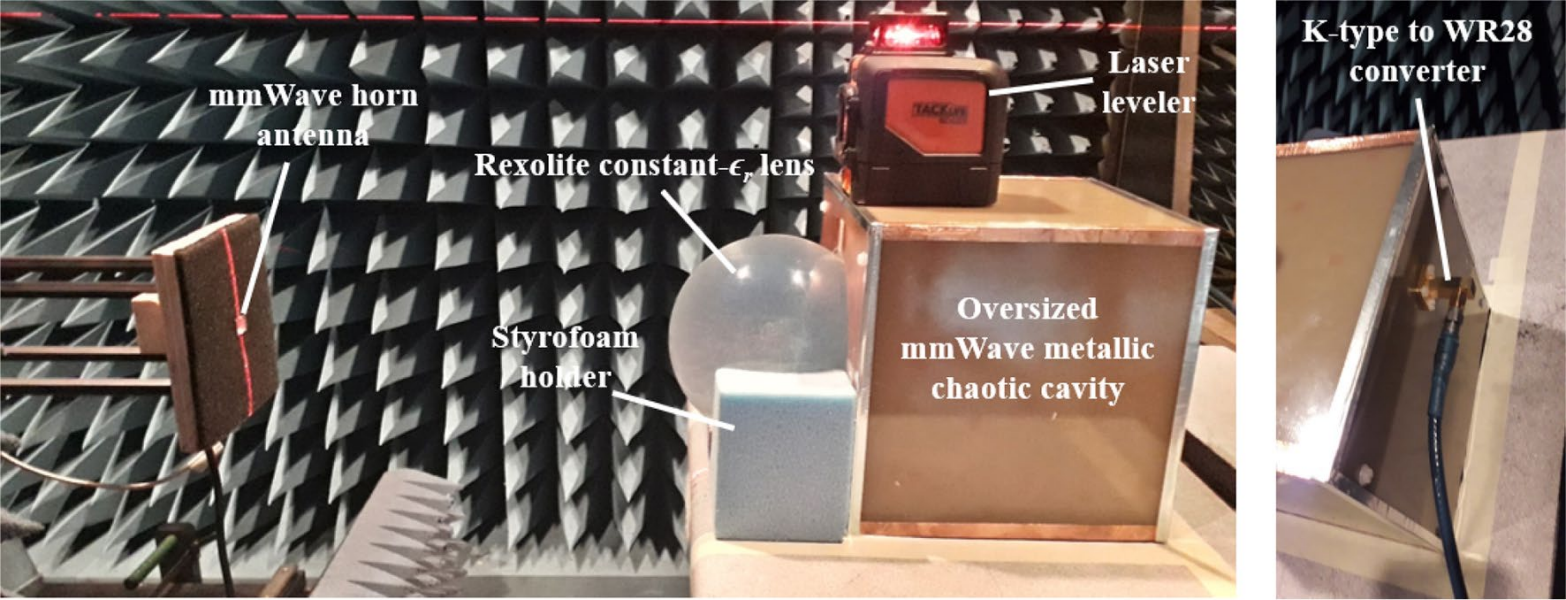
Lens-loaded cavity hardware and measurement setup in a near-field anechoic chamber.

**Figure 7:**
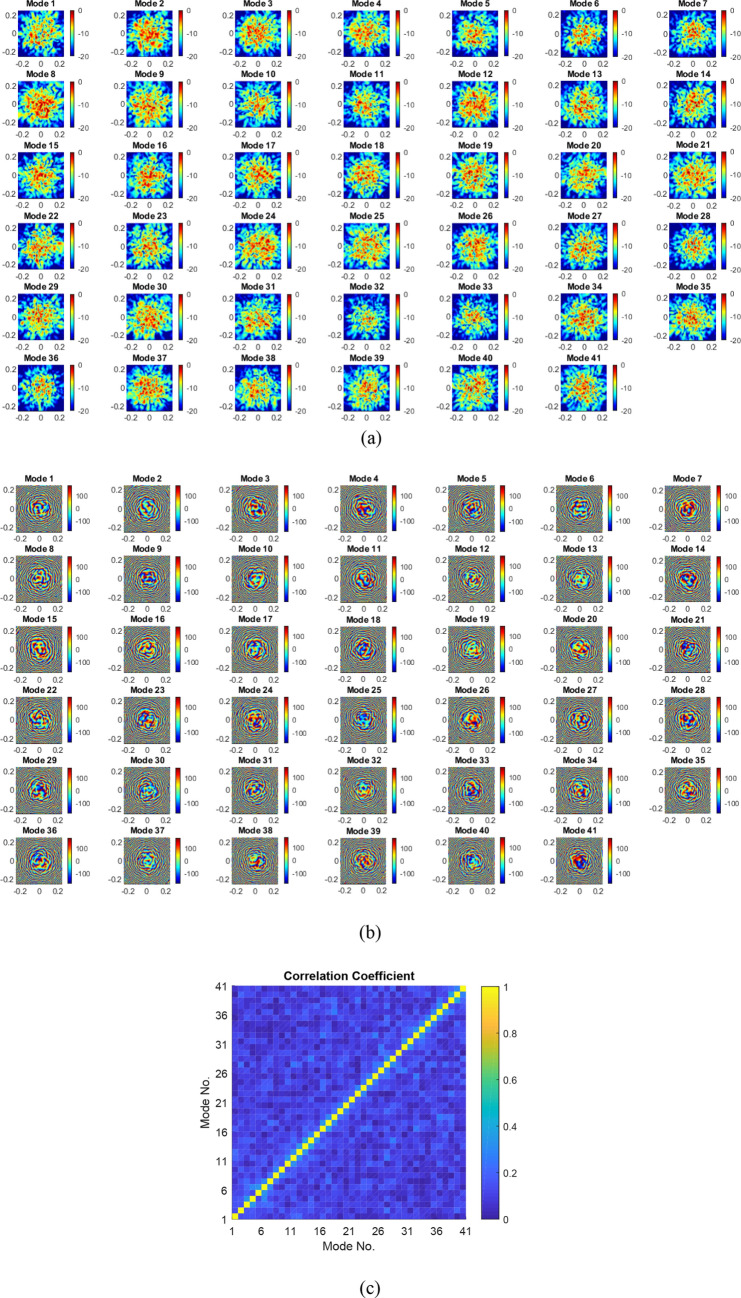
Measured near-field contour plots (**a**) magnitude, (**b**) phase, (**c**) correlation coefficient surface plot for *N* = 41 modes.

**Figure 8:**
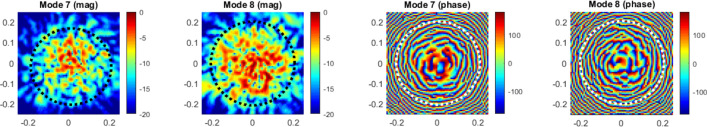
Comparison between magnitude and phase contours of mode 7 and 8.

The entire assembly is placed in a planar NSI near-field anechoic chamber where a mmWave horn antenna operating from 27 to 29 GHz is used to record the field on a plane 0.5 m from the lens-loaded cavity. The fabricated lens-loaded cavity and the measurement setup is shown in Fig. 6. Separate measurements were taken in the vertical and horizontal planes and the results in the form of magnitude and phase of *N* = 41 modes are given in Fig. 7a,b. The field peaks are randomly projected, and the sectoral coverage provided by the lens-loaded cavity is evident from the magnitude plots, as predicted, Fig. 7a. Lowering the mode number is the key to reduce the computational complexity of the retrieval problem, as well as calibration over frequency for each operating mode. We now show how this is possible, thanks to the fact that the measurement modes are highly orthogonal due to the presence of the lens. This is evident from the correlation between modes shown in Fig. 7c. In addition to this, by using fewer number of points to sample the frequency band (i.e. *N* = 41 is much lower than *N*_max_ = 331), we are increasing the frequency interval between our measurement modes, further de-correlating the measurements. A detailed version of the phase plots in Fig. 8 further indicate that mode symmetry is broken, which is beneficial for accurate DoA estimation as it reduces the chances of ghost DoA estimation. The reason behind the requirement of symmetrical irregularities in the modes to avoid ghost images can be found in^42^. Consider, for example, for a radiation source at *θ* = 0° and *φ* = 10°, there might be a chance of ghost DoA estimation at *θ* = 0° and *φ* = − 10° in case of symmetrical phase response, which is not the case in the lens-loaded cavity measured phase response shown in Fig. 8. In^42^ the coded apertures are positioned in an irregular manner in order to break the regular periodicity, while in this study, a metallic scatterer internally positioned in the cavity is mainly responsible for breaking the mode symmetry.

## DoA estimation from measurement modes

The DoA estimation problem is an estimate of the plane-wave projection pattern on the characterization plane, which can be analysed from the compressed measurement as follows^24^:

7 $$p_{est,M} = E_{M \times N}^{\dag } g_{N}$$

where the lens-loaded cavity characteristic aperture conjugate transposed (donated by (·)$^{\dag }$) transfer function is applied to the compressed measurement (also known as matched-filtering). The number of modes is denoted by *N* and the number of pixels on the characterization plane is denoted by *M*. Note that we use plane wave projection model which accurately mimics real world signal. As in^24^, to minimize the error residue in the estimation of the objective function, we use the least-squares reconstruction iterative algorithm given as

8 $$p_{est + 1,M} = \arg \min \left\| {g_{N} - E_{N \times M} p_{est,M} } \right\|_{2}^{2}$$

**Figure 9:**
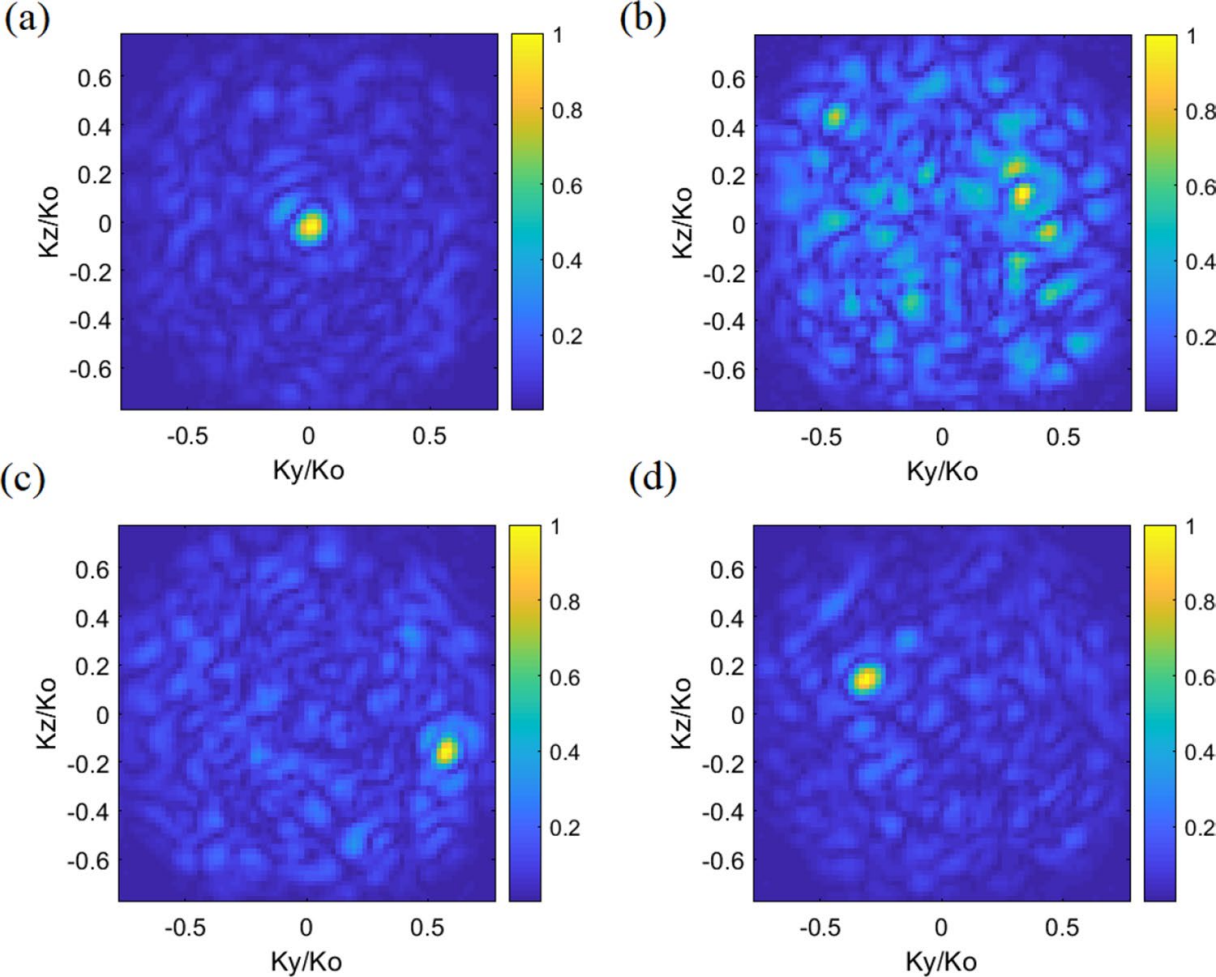
DoA estimation using near-field measured results of 41 modes from 27.0 to 29.0 GHz when a plane wave at 28 GHz strikes the lens-loaded cavity: (**a**) Case 1: (*θ* = 0°, *φ* = 0°), (**b**) Case 2: (*θ* = 20°, *φ* = 20°), (**c**) Case 3: (*θ* = 35°, *φ* = − 15°), and (**d**) Case 4: (*θ* = 20°, *φ* = − 20°) at SNR = 20 dB.

It is evident in (8) that the least-squares solution is an iterative process, making use of the matched-filter solution in (7) as an initial estimate. After the retrieval of source projection pattern estimation, the DoA estimation can be directly achieved by a Fourier transform operation applied to the evaluated $p_{est}$, while the incident angles in *θ*, *φ* can be retrieved using peak finding algorithm from the patterns. Following this approach using the measurements of the lens-loaded cavity, we successfully evaluated the DoA for 4 sample cases given in Fig. 9 while the comparison between the evaluated DoA and the ground truth is provided in Table 1. Note that for the reconstructions provided in Fig. 9, 20 dB Gaussian noise is added to the measurements, according to^30^:

9 $$g_{n} = g + n\left( \sigma \right)$$

where *g*_*n*_ is the measurement with the added noise and *n*(*σ*) is the Gaussian identical independent distribution (i.i.d) noise model with zero mean and variance of *σ*^2^ = SNR|*g*|. The noise is defined with respect to the average received signal over all frequency samples, |*g*|. As indicated in previous section, although a very high number of measurement modes is theoretically desirable for a frequency-diverse aperture antenna to work, reliable DoA estimation is practically possible using only 41 modes with the proposed lens-loaded cavity structure.

**Table 1:** Estimated DoA values in comparison to the ground truth at SNR = 20 dB.

Case	Ground truth	Estimated
1	*θ* = 0°, *φ* = 0°	*θ* = 0°, *φ* = 0°
2	*θ* = 20°, *φ* = 20°	*θ* = 18.5°, *φ* = 20.2°
3	*θ* = 35°, *φ* = − 15°	*θ* = 36°, *φ* = − 15.1°
4	*θ* = − 20°, *φ* = − 20°	*θ* = − 19.5°, *φ* = − 23°

The modes exploitable in our lens-loaded cavity correspond to independent degrees of freedom used to reconstruct the interrogated space. The problem is relaxed compared to a classical imaging system because the depth is not probed, restricting the dimensions of the unknowns to the transverse components of the plane wave vectors. Under the conditions of this experiment, it is possible to reconstruct a maximum of 41 independent pieces of information related to the DoAs of the incident signals, making the lens-loaded cavity a good candidate for mmWave channel sounding applications at a reasonable SNR level^30^.

**Figure 10:**
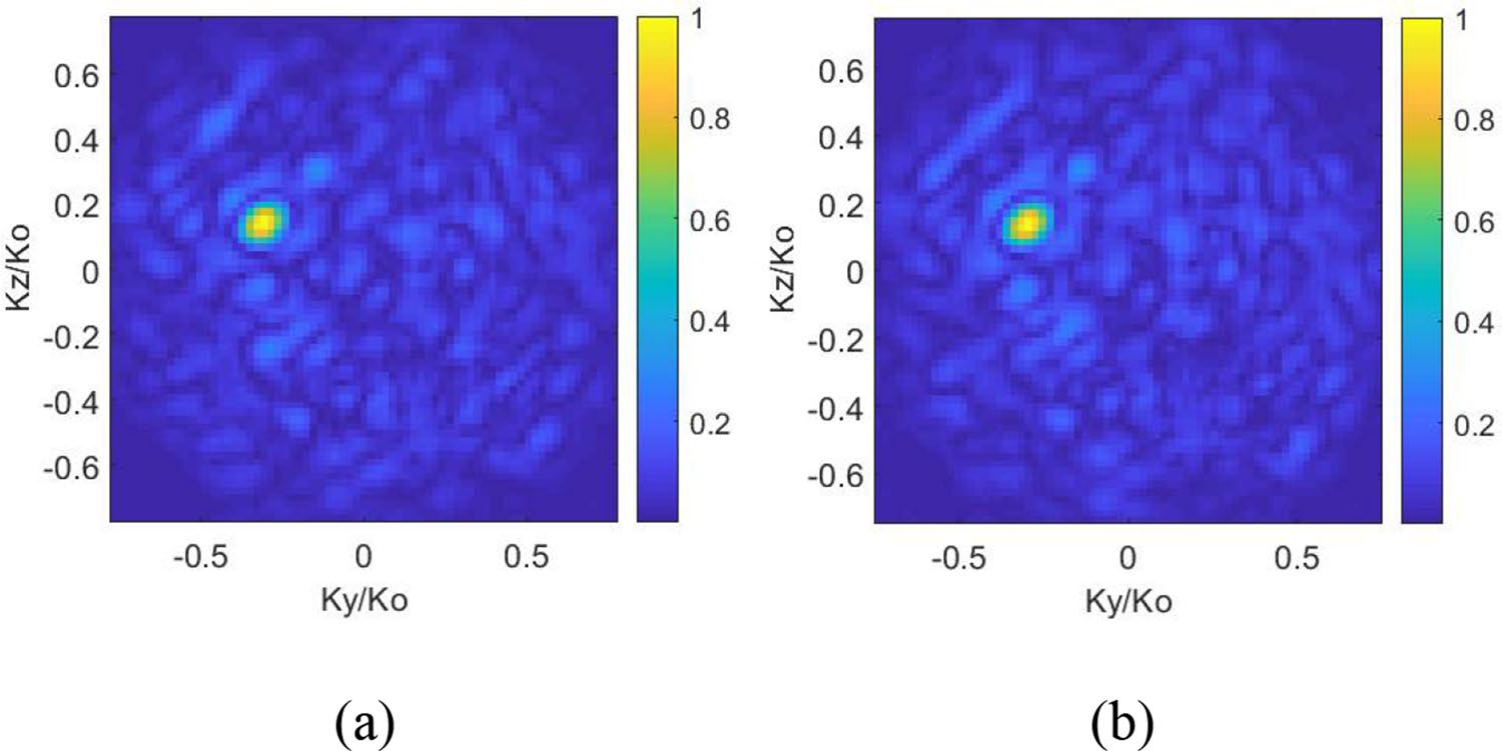
Retrieved DoA patterns of case 4: (*θ* = 20°, *φ* = − 20°) (**a**) when the far-field source spectrum is frequency independent, i.e. 28 GHz, (b) when the far-field source is frequency-dependent (27–29 GHz).

One of the assumption made in this work is that the power spectrum of the source field incident upon the lens-loaded cavity does not vary as a function of frequency. Please note, this is not a fundamental limitation of the presented technique, but is an assumption to simplify the mathematical model of the far-field source. The fractional bandwidth of the studied 5G channel is around 7% and the assumption that the spectrum of the incident plane-wave remains constant can be justified by the much higher frequency of the plane-wave, 28 GHz, with respect to the variation across center frequency, ± 1 GHz. To elaborate this, we take one of the studied scenarios (case 4: *θ* = 20°, *φ* = − 20°) and model the incident plane-wave, *P* in (5), as frequency-independent (28 GHz) and frequency dependent (between 27 and 29 GHz). Comparing the reconstructed DoA estimation patterns in the Fig. 10, it can be seen that this assumption does not have a significant effect in the reconstruction.

**Figure 11:**
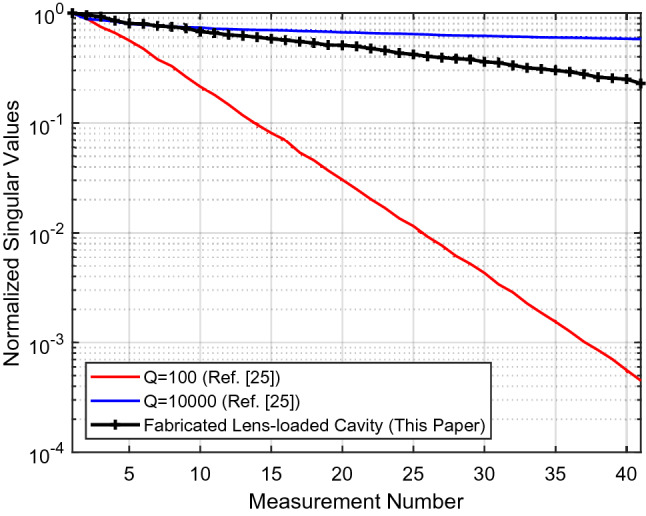
SVD contours for the physical lens-loaded cavity.

SVD is a measure of diversity of the field pattern generated by frequency-diverse cavities while looking towards a coverage area. The mode orthogonality enhancement discussed in the previous section SVD due to the presence of spherical constant-*ϵ*_*r*_ lens has an implication in the SVD contour, as can be seen from Fig. 11. General form of the SVD can be written as^29^

10 $$E_{N \times M} = U_{N \times N} S_{N \times M} V_{M \times M}^{T} ,$$

when *U* and *V* are unitary matrices and *S* is a rectangular diagonal matrix with the singular values in descending order, while (·)^*T*^ is the matrix transpose operation. The *Q* = 100 and *Q* = 10,000 patterns corresponds to the analytical approach in^24^ with same number of measurement modes, 41, in comparison to the fabricated lens-loaded cavity. The Q-factor of the ideal model is, approximately 4600, and the SVD result obtained from the experimental cavity in Fig. 11 remains inside the region bounded by *Q* = 100 and *Q* = 10,000 patterns calculated in^24^ within the same frequency band of operation, 27–29 GHz. As the number of modes increase, the normalized singular values decrease, but not drastically, ensuring that the proposed lens-loaded cavity modes have a reasonable orthogonality. The Q-factor of the fabricated cavity can be further enhanced by carefully exploring the mmWave power coupling mechanism and reducing the coupling parameter and coupling coefficient. This can be done by further investigating coupling techniques other than the ones used in the paper, e.g. probe, loop or modified open guide coupling etc.

## Conclusion

In this paper, we have presented a novel structural configuration of a lens-loaded cavity operating as a frequency-diverse antenna created using an oversized mmWave chaotic cavity and a spherical constant-*ϵ*_*r*_ lens for DoA estimation. A proof-of-concept lens-loaded cavity is developed using a metallic cavity and a Rexolite spherical constant-*ϵ*_*r*_ lens, operating in 27–29 GHz mmWave 5G frequency bands. The presented lens-loaded cavity captures the channel information and compress the incoming plane wave source patterns into a single channel, which requires only a single RF chain hardware to successfully retrieve the DoA information, resulting in aggressive hardware reduction compared to classical DoA estimation methods. A set of near-field measurements in vertical and horizontal planes are taken in anechoic chamber and computational methods are used with the measured data to retrieve arbitrary far-field radiation source information from which a plane wave in mmWave frequency bands strikes the lens-loaded cavity. Although demonstrated for the DoA estimation of individual far-field sources, our initial studies also suggest that the presented technique can be scaled to the DoA estimation of multiple sources and assigned with independent phase references. This aspect of the presented computational DoA system will be pursued in future works. For the spherical constant-*ϵ*_*r*_ lens development, we use high quality Rexolite material and ensured controlled experimental work, however, other low-cost low-loss plastic materials like poly (methyl methacrylate) or PMMA can be used for the lens development. The primary application of the proposed approach is mmWave channel sounding for 5G and beyond, while extension of the same system architecture can find further applications in smart antenna systems, navigation systems, radar tracking, mmWave communication, and radio astronomy.
